# Asymmetrical Split-and-Recombine Micromixer with Baffles

**DOI:** 10.3390/mi10120844

**Published:** 2019-12-03

**Authors:** Wasim Raza, Kwang-Yong Kim

**Affiliations:** Department of Mechanical Engineering, Inha University, Incheon 22212, Korea; wasimkr@live.in

**Keywords:** micromixers, unbalanced split-and-recombination, baffles, Navier–Stokes equations, parametric study, mixing index

## Abstract

The present work proposes a planar micromixer design comprising hybrid mixing modules of split-and-recombine units and curved channels with radial baffles. The mixing performance was evaluated numerically by solving the continuity and momentum equations along with the advection-diffusion equation in a Reynolds number range of 0.1–80. The variance of the concentration of the mixed species was considered to quantify the mixing index. The micromixer showed far better mixing performance over whole Reynolds number range than an earlier split-and-recombine micromixer. The mixer achieved mixing indices greater than 90% at *Re* ≥ 20 and a mixing index of 99.8% at *Re* = 80. The response of the mixing quality to the change of three geometrical parameters was also studied. A mixing index over 80% was achieved within 63% of the full length at *Re* = 20.

## 1. Introduction

The adoption of various microfluidic devices has advanced very fast and effectively in the field of chemical synthesis, biochemical analysis, biomedical diagnostics, and drug delivery [1,2,3]. The advantages of microfluidic systems include reduced consumption of reagent and sample volumes, fast sample processing, low cost, better heat and mass transfer efficiency, improved portability, and scalability [4]. Mixing of two or more species is one of the fundamental processes that must be achieved for most of the microfluidic applications, such as biological and chemical analyses.

The flow regime in microfluidic devices corresponds to laminar flow because of the micro size of the channel. Mixing relies on diffusive transport because of the absence of turbulence in the low Reynolds number range. However, mixing through molecular diffusion is too slow and ineffective for most of the envisioned microfluidic applications. Hence, it is critical to develop efficient micromixers for the progress of microfluidic systems.

Passive and active micromixing are the two categories of approaches employed to enhance mixing. The flow behavior in passive micromixers is self-governing and relies on only the geometrical structure of the microchannel to agitate the flow and accelerate mixing. In contrast, active micromixers use external stimuli in the form of electric [5], acoustic [6], and magnetic [7] disturbances to regulate the flow. Although the active micromixers have better mixing performance, the requirement of additional perturbation actuators and energy sources limits their use in microfluidic systems. Thus, the simple manufacturing methods and uncomplicated assembly make the passive micromixers more preferable [8,9].

Chaotic advection and lamination [8] lead to the efficient homogenization of fluid species in passive micromixers. Micromixers that employ the lamination process utilize the molecular diffusion through the interface between fluid layers. Hence, an increase in the interfacial surface and a reduction in the diffusion path enhance the mixing in a lamination-based micromixer. The main channel fluid steam is split into several streams and then rejoined to build a laminated stream.

Hirata and Ohkawa [10] experimentally tested a split-and-recombine (SAR) micromixer composed of three-dimensional (3D) bent channels that produce deformation and rotation of the fluid interface, which were visualized using a laser-induced fluorescence method. A combination of 3D channels with bending angles of 120° and 60° produced a 180° rotation of the fluid interface. Chen et al. [11] performed mixing and flow analyses on a cascaded SAR micromixer at *Re* = 34.6–150. Triangular baffles were arranged asymmetrically to execute split and recombination, and secondary flows in the form of the corner and Dean vortices were generated through the sharp bend of the baffles, which produced chaotic advection and improved the mixing quality.

Advection refers to the transport mechanism resulting from the bulk motion of fluids. In lamination-based micromixers, advection occurs in the direction of the flow, so it does not aid in the mixing. However, chaotic advection occurs in the transverse direction and extends the interfacial area exponentially, thereby enhancing the mixing quality. Transverse flows can be produced by the use of baffles [12,13], staggered herringbone grooves [14], 3D channels [15,16], and two-dimensional (2D) cured channels [17,18].

Ansari and Kim [17] used the SAR mechanism and secondary transversal flows by means of Dean vortices in the curved sub-channels of a micromixer to obtain a mixing index of 0.52 at *Re* = 80 [17]. The recombination of the fluids at the junctions of the sub-channels produced unbalanced collision because of the unequal width of the sub-channels, and the Dean vortices in the curved channels deformed the interfaces to enhance the mixing. Tsai and Wu incorporated baffles in a 2D curved channel to utilize multidirectional vortices for mixing in a micromixer [13]. The micromixer showed good concentration uniformity at low and high Reynolds numbers only.

Various microfluidic applications involve different flow conditions [19]. Hence, homogeneity of the species over a wide range of flow conditions is preferable. To achieve this, there has been a recent trend of combining different mixing mechanisms [16,20]. Bazaz et al. [20] numerically and experimentally analyzed a planar hybrid micromixer consisting of an obstruction, nozzle, pillar, and Tesla units. The improved performance with a mixing index over 90% for *Re* = 0.001–0.1 and 22–45, suggested that the assembly of different 2D mixing units is an effective strategy. Recently, several complicated 3D micromixers achieved extremely high mixing performance [16], but 2D passive micromixers still have advantages in fabrication and integration into micro devices.

Earlier research indicates that the unbalanced inertia of streams flowing through sub-channels of unequal widths can significantly promote mixing [17,21]. However, the non-presence of the two fluids together in some of the sub-channels of these designs [17,21] affects the fluid interface diffusion and mixing. It is evident from the literature that baffles in a curved channel produce vortices in the longitudinal and transverse directions that promote mixing [13]. Hence, by combining these two mixing mechanisms, the present study proposes a micromixer with curved microchannels with radial obstructions and unbalanced split-and-recombine (SAR) units. The mixer demonstrates excellent fluid homogeneity in the micromixer over a broad range of flow conditions. The flow and mixing capability were investigated numerically in a Reynolds number range of 0.1–80. The variation of the mixing quality was also studied with the change in the geometric parameters at different Reynolds numbers.

## 2. Micromixer Model

**Figure 1:**
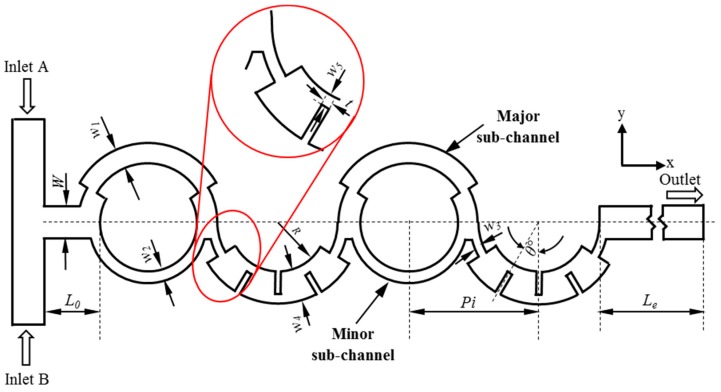
A diagram of the proposed micromixer.

Figure 1 presents the layout of the proposed micromixer. The layout consists of a combination of split-and-recombination and curved microchannel with radial baffles as mixing units. The primary microchannel has a rectangular cross-section of 300 µm × 200 µm and splits into sub-channels with asymmetrical widths. The width of the narrower sub-channel (*w*_2_) is half the width of the broader sub-channel (*w*_1_), which is the optimized ratio of sub-channel widths proposed by Ansari and Kim [17]. The two sub-channels together form a circular shape. At the recombination of the first unit, a curved microchannel with a radial obstruction protruding from the wall is connected. The geometrical parameters of the proposed micromixer and their reference values are listed in Table 1.

The mechanisms anticipated for mixing include Dean vortices in the curved channels, vortices in the longitudinal plane because of the baffles, and unequal inertial collision because of disproportionate SAR. The chaotic advection will extend the fluid interfacial area exponentially and decrease the diffusion length, which enhances mixing [22,23].

## 3. Analysis Methods

The flow inside the microchannel was assumed to be steady, incompressible, laminar, and Newtonian. The continuity (Equation (1)), momentum (Equation (2)), and convection-diffusion (Equation (3)) equations were solved numerically for the flow and mixing analysis. The CFD software ANSYS CFX 15.0^®^ (ANSYS Inc., Canonsburg, PA, USA) [24] employs a coupled solver and finite volume technique to discretize Equation (1)–(3):

(1) $$\nabla \cdot \vec{V}=0$$

(2) $$\left(\vec{V}\cdot \nabla \right)\vec{V}=-\frac{1}{\rho }\nabla P+\nu \nabla^{2}\vec{V}$$

(3) $$\left(\vec{V}\cdot \nabla \right)C=D\nabla^{2}C$$

where $\vec{V}$, *C*, *D*, *ρ*, *P*, and *ν* are the velocity, dye concentration, diffusion coefficient, density, pressure, and kinematic viscosity, respectively.

Water and a dye-water solution, which both have the physical properties of the water, enter the two inlets. The density, diffusivity, and dynamic viscosity used in the simulation were 997 kg/m^3^, 1.2 × 10^−9^ m^2^/s, and 8.9 × 10^−4^ kg·m^−1^·s^−1^, respectively [25]. Zero-velocity at the solid walls, uniform inlet velocity, and atmospheric pressure at the outlet were set as the boundary conditions. The molar concentrations at inlets A and B were assigned values of 1 and 0, respectively. The inlet velocity corresponds to the Reynolds number depending on the hydraulic diameter of the inlet channel.

Artificial diffusive fluxes are caused by the discretization of convective terms, and the complete elimination of these numerical errors is not possible. However, they can be reduced by adopting high-order discretization schemes such as a second-order upwind scheme and or third-order QUICK schemes [26]. In the present study, the second-order upwind scheme was used for the convection terms as a high-resolution approximation technique [24]. The convergence criterion was a value less than 1.0 × 10^−6^ for the root-mean-squared residual of each variable.

The mixing index at any transverse plane can be calculated as follows [27]:

(4) $$M=1-\frac{\sigma }{\sigma_{\max}}$$

(5) $$\sigma =\sqrt{\frac{1}{N}\overset{N}{\underset{i=1}{\sum }}{\left(c_{i}-{\bar{c}}_{m}\right)}^{2}}$$

where *σ*, $\sigma_{\max}$, *N*, *c_i_*, and ${\bar{c}}_{m}$ are the standard deviation, maximum standard deviation of the dye concentration, number of samples on the plane, concentration at the chosen point *i*, and average *c_i_*, respectively. The number of samples (*N*) to calculate the mixing index at the exit was selected as 150 × 100. A smaller value of the standard deviation will produce more significant homogeneity of the mixing species. Hence, completely segregated fluids show *M* = 0, while entirely homogenous fluids show *M* = 1.

## 4. Results and Discussion

**Figure 2:**
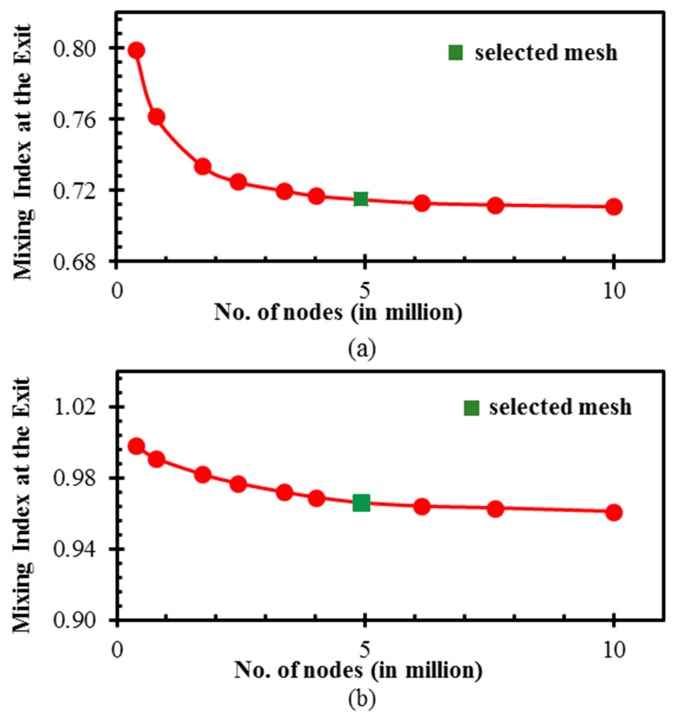
Grid refinement tests at: (**a**) *Re* = 0.1 and (**b**) *Re* = 40.

The spatial discretization of the micromixer domain was done by using a hexahedral grid system. A comprehensive grid dependency analysis was done to find the optimum grid system at *Re* = 0.1 and 40, which ensures the grid-independency of the results. The analysis was performed for the mixing index at the exit in a wide range of node numbers of 3.82 × 10^5^ to 10.00 × 10^6^. Based on a compromise between accuracy and computational time for the calculation, an optimum grid system with 4.92 × 10^6^ nodes was chosen from the results shown in Figure 2. The computation times with the finest and optimum grids at *Re* = 40 were 62.5 and 3 hours, respectively, on a central processing unit (CPU) with a 3.4-GHz Intel Core *i*7, 8 cores, and 32 GB of random access memory (RAM). The difference in the mixing index at the outlet between the optimum and the finest grids was trivial (0.55% and 0.57% at *Re* = 0.1 and 40, respectively).

The grid convergence index (*GCI*) based on Richardson extrapolation was also analyzed to quantify the discretization uncertainty of the grid system at *Re* = 40. The mixing index at the exit was selected as a reference parameter. The detailed procedure of the *GCI* study can be found in the works of Roache [28,29] and Celik and Karatekin [30]. Numerical simulations were performed to evaluate the mixing index using three different grid systems with *N*_1_ = 4.65 × 10^6^ cells, *N*_2_ = 3.15 × 10^6^ cells, and *N*_3_ = 1.56 × 10^6^ cells. The grid refinement factor and the factor of safety were 1.3 and 1.25, respectively. The discretization errors are listed in Table 2. The accuracy of the simulation increases with the mesh refinement, as indicated by the reduction in *GCI* from 1.92% to 1.16%. Furthermore, if the ratio *GCI*_2,3_/(*r_p_*·*GCI*_1,2_) is closer to unity (0.992 in the present case), the results are within an asymptotic range of convergence, and further refinement is not required. Hence, the grid with 4.92 × 10^6^ nodes corresponding to *N*_1_ = 4.65 × 10^6^ cells was selected for further calculations.

**Figure 3:**
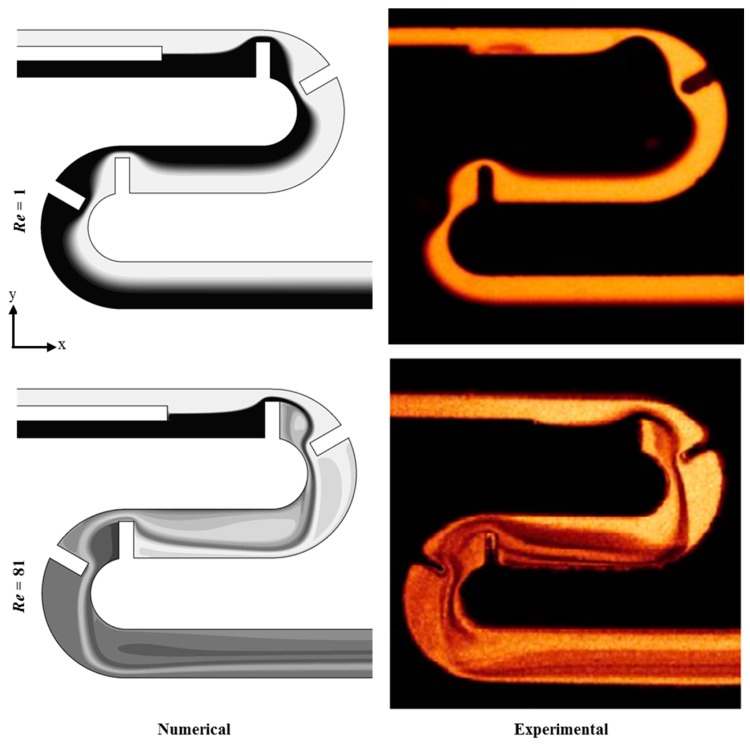
Comparison of numerical results with experimental images of Tsai and Wu [13] for concentration distribution. Reproduced with permission from [13].

Before the estimation of the homogenization capability of the proposed micromixer, qualitative validation of the numerical model was performed by comparing the results with experiment results obtained by Tsai and Wu [13]. Experimental confocal images of the concentration distribution on the horizontal mid-plane were compared with numerical plots obtained at *Re* = 1 and 81, as shown in Figure 3. There was acceptable similarity between them, which instills confidence in the numerical results.

**Figure 4:**
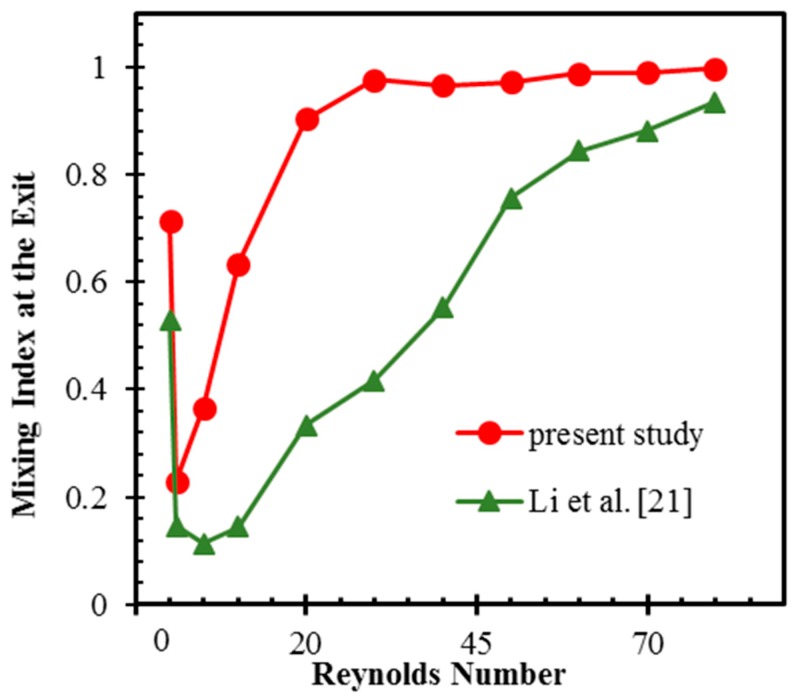
Comparison of mixing index of the proposed micromixer with the planar asymmetrical split-and-recombine (P-ASAR) micromixer [21].

Among the 2D micromixers that utilize unequal mass flux collision of fluids, the planar asymmetrical SAR (P-ASAR) micromixer [21] has shown the best mixing capability. Therefore, its mixing quality was compared with that of the proposed micromixer at several Reynolds numbers between 0.1 and 80, as presented in Figure 4. The proposed micromixer shows higher mixing quality over the whole range of Reynolds numbers. A transition of mixing from diffusion to chaotic dominant takes place at *Re* = 1 in the proposed micromixer, while it takes place at *Re* = 5 in the P-ASAR micromixer. At *Re* = 5, the proposed micromixer shows a 221% higher mixing index than the P-ASAR micromixer.

The capacity to homogenize fluids in the proposed micromixer increases more rapidly with the Reynolds number. The proposed micromixer reaches mixing indices over 90% at Reynolds numbers larger than 20, while the P-ASAR micromixer reaches a similar mixing at a Reynolds number of 80. The proposed micromixer achieves an extremely high mixing index of 99.8% at *Re* = 80. At *Re* = 20, the P-ASAR micromixer shows a 63% lower mixing index than the proposed micromixer. The P-ASAR micromixer attains a mixing index of 84.4% at a Reynolds number of 60, which is still 14% less than that of the proposed micromixer.

**Figure 5:**
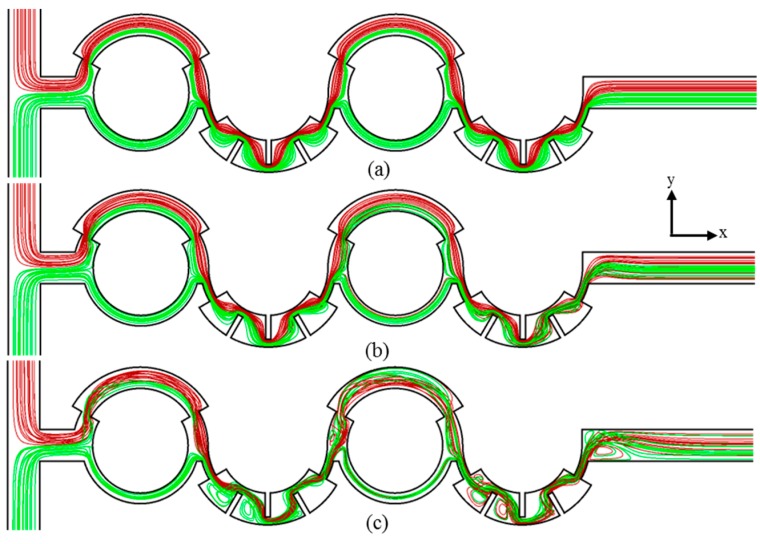
Projected streamlines in the micromixer: (**a**) *Re* = 0.1; (**b**) *Re* = 5; and (**c**) *Re* = 20.

**Figure 6:**
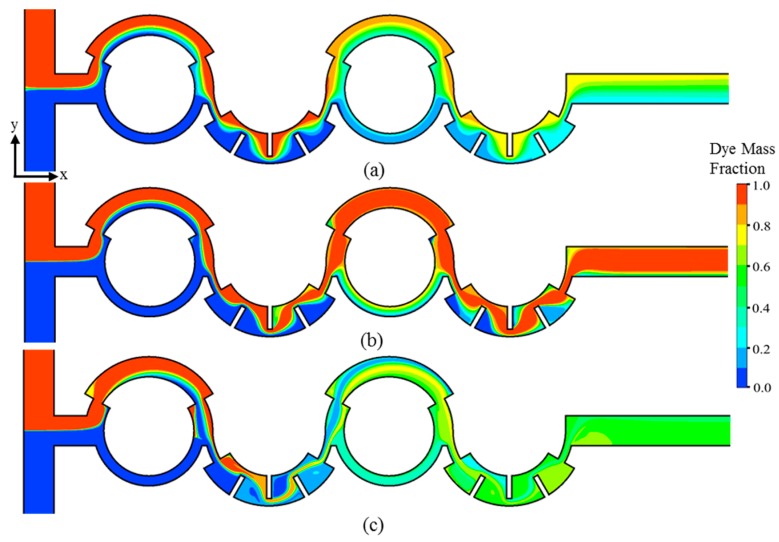
Concentration distribution in the micromixer: (**a**) *Re* = 0.1; (**b**) *Re* = 5; and (**c**) *Re* = 20.

Figure 5 and Figure 6 show the projected streamlines of fluids entering from the two inlets and the concentration distributions on the horizontal plane at the channel half-depth. These results were used to investigate the flow patterns that assist in mixing at *Re* = 0.1, 5, and 20. The two fluids meet at the T-joint, the single stream is split into two streams in the first mixing unit with two sub-channels of unequal widths. The streams recombine at the collision zone, and the combined stream flows through the second mixing unit with radial obstructions. The bending of streamlines because of the staggered arrangement of the baffles causes elongation of fluid interfaces. These processes are repeated in the next two mixing units.

At low *Re* = 0.1, the two fluid streams flow side by side, and there is no lateral advection, as shown in Figure 5a. Hence, the mixing takes place by diffusion only. The radial obstacles bend and distort the fluid interfaces to expand the interfacial area for diffusion, thereby promoting mixing, as presented in Figure 6a. At *Re* = 5, the mixing shows an initial stage of transition from pure diffusion to chaotic advection. At this Reynolds number, the streamlines start to flip as the flow passes through the baffles in the second mixing unit, which enhances the chaotic advection, as shown in Figure 5b. However, the higher velocity at *Re* = 5 than at *Re* = 0.1 reduces the residence time of the fluids, which reduces mixing overall, as shown in Figure 6b.

At *Re* = 20, the streamlines are no longer parallel and begin to flip in the first mixing unit because of the change in the cross-sectional area in the major sub-channel. The onset of recirculation and twisting of the flow is visible behind the baffles in the second mixing unit, as shown in Figure 5c. The twisting and flipping of streamlines along with flow recirculation give rise to chaotic advection, which expands the area of the fluid interface by stretching and folding it to enhance mixing. Furthermore, the passage between the baffles and the wall increases the local flow velocity and enhances chaotic advection. An almost uniform concentration distribution is achieved at *Re* = 20, as presented in Figure 6c.

**Figure 7:**
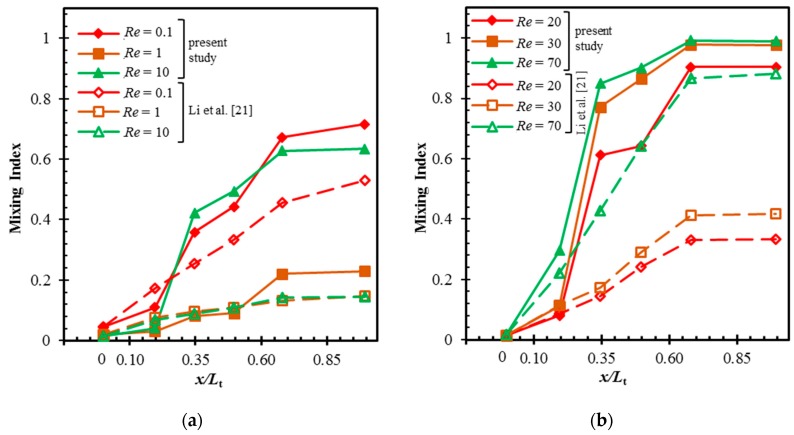
Comparison of developments of mixing index along the length between the proposed micromixer and that of Li et al. [21] at different Reynolds numbers: (**a**) *Re* = 0.1–10 and (**b**) *Re* = 20–70.

Figure 7 presents the progress of mixing at different Reynolds numbers inside the proposed micromixer and P-ASAR micromixer [21]. The mixing indices were estimated on the y-z planes at the main channel inlet, the junctions between mixing units, and the outlet of the channel at several Reynolds numbers. The mixing develops a higher rate at *Re* = 0.1 than at Reynolds numbers of 1 and 10 for both micromixers. The mixing indices in the proposed micromixer are similar at *Re* = 0.1 and 10, while the mixing index at *Re* = 0.1 is much higher than that at *Re* = 10 in the P-ASAR micromixer [21]. This can be attributed to the enhanced chaotic advection in the proposed micromixer at *Re* = 10, which compensates for the reduction in diffusive mixing because of the shorter residence time at higher Reynolds number.

The development rate of the mixing index in the proposed micromixer increases with the increase in Reynolds number from 1 to 10 because of the onset of chaotic advection at *Re* = 5, as shown in Figure 5. In contrast, the development rate in the P-ASAR micromixer [21] at *Re* = 10 is similar to that at *Re* = 1. The rates of increase in the mixing index through the second and fourth mixing units are higher than those of the first and third mixing units in the proposed micromixer. However, the mixing develops steadily through the mixing units in the P-ASAR micromixer [21]. This confirms the advantages of introducing the mixing units with radial baffles that promote the combination of various mixing mechanisms. The proposed micromixer attains a mixing index of more than 0.80 within 63% and 40% of the total length at *Re* =20 and *Re* ≥ 30, respectively. The P-ASAR micromixer [21] achieves a mixing index over 0.8 within 63% of the full length at a much higher Reynolds number of 70.

**Figure 8:**
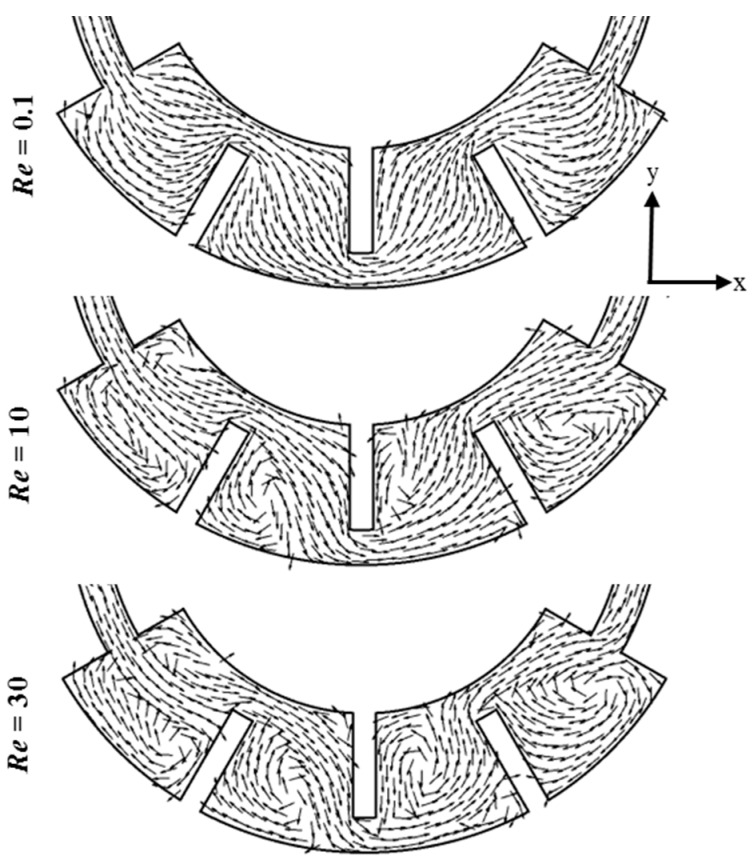
Velocity vectors on the x-y plane at half channel depth in the second mixing unit of the proposed micromixer at different Reynolds numbers.

Figure 8 shows the velocity vectors on the x-y midplane in the second mixing unit of the proposed micromixer. There is a distinct effect of the Reynolds number on the flow structure. At *Re* = 0.1, the fluids flow along the wall, and no vortices are observed on the horizontal plane. However, at *Re* =10, the onset of separation vortices occurs because of the baffles. At *Re* = 30, the area covered by separation vortices downstream of the baffles increases. Concerning unbalanced collisions, previous studies [17,21] observed that Dean vortices and unbalanced collisions of the fluid streams are useful for mixing at high Reynolds numbers. Hence, the merging of asymmetrical recombination, separation vortices, and Dean vortices enhance the mixing in the proposed micromixer.

**Figure 9:**
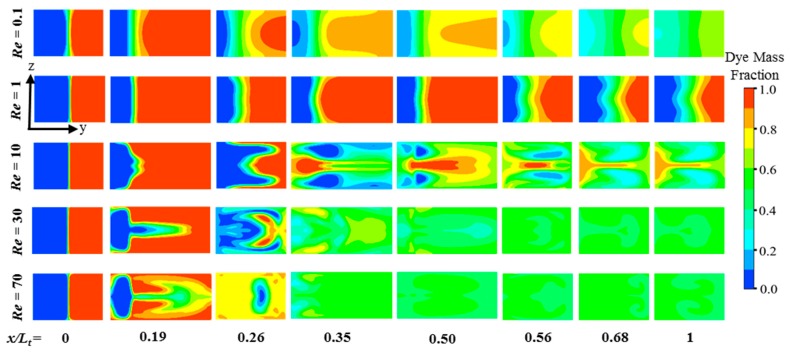
Dye mass fraction distributions on the cross-sectional planes along the length for *Re* = 0.1, 1, 10, 30, and 70.

Figure 9 shows the distributions of dye concentration on the transverse planes at different axial positions along the length of the micromixer at *Re* = 0.1, 1, 10, 30, and 70. There is slight distortion of the interface on the planes at *x/L_t_* = 0.26 (the intermediate baffle in the second unit), 0.35 (the end of the second unit), 0.56 (the intermediate baffle in the fourth unit), and 0.68 (the end of the fourth unit)). Nevertheless, there is no notable manipulation of fluid interface at *Re* = 0.1 and 1, and the diffusion is the only mechanism responsible for the mixing. Furthermore, enhanced distortion of the fluid interfaces is observed at a higher Reynolds number of 10, which increases the interfacial area for enhanced mixing. More substantial distortions of the fluid interface occur on the planes around the baffles (*x/L_t_* = 0.26, 0.35, 0.56, and 0.68), which are responsible for the enhanced development rate of mixing in this zone, as indicated in Figure 7. The distortion of the fluid interface becomes more active as the Reynolds number increases (*Re* = 30 and 70), which causes an earlier establishment of a uniform distribution of mixing species on the planes.

**Figure 10:**
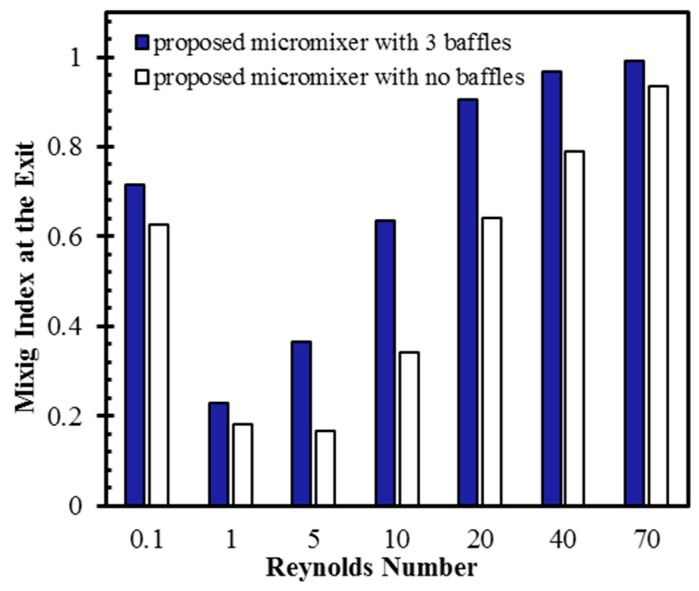
Effect of baffles on the mixing capability of the micromixer for different Reynolds number.

To understand the effect of the baffles on mixing in the proposed micromixer, its mixing index was compared with that of a micromixer without baffles, as presented in Figure 10. At *Re* = 0.1, 1, and 70, there are relatively insignificant differences between the mixing qualities of the micromixers with and without baffles. The micromixer with baffles still shows higher mixing indices than the micromixer without baffles. At *Re* = 5, the proposed micromixer shows a 120% higher mixing index than the micromixer without baffles. This demonstrates the early transition of the mixing mechanism from diffusion to chaotic advection in the proposed micromixer because of the baffles, as shown in Figure 4. There were increases of 85%, 41%, and 23% in the mixing index of the proposed micromixer in comparison to the micromixer without baffles at *Re* = 10, 20, and 40, respectively.

**Figure 11:**
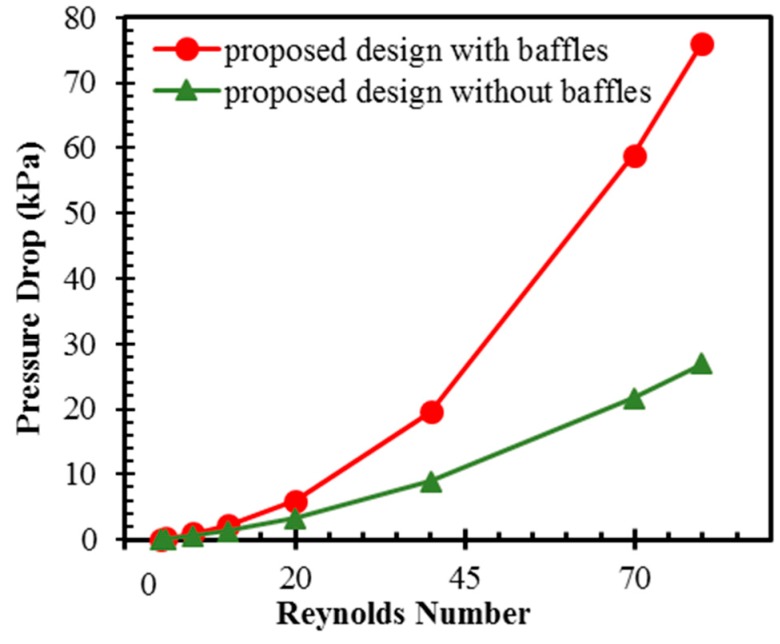
Variations of pressure drop with Reynolds number in the proposed design with and without baffles.

Figure 11 shows the pressure drop as a function of the Reynolds number in the proposed micromixer with and without baffles. As expected, the pressure drop in the proposed micromixer with baffles is much higher than that in the micromixer without baffles. The difference in pressure drop between these two designs rises with Reynolds number and becomes significant at high Reynolds numbers, as shown in Figure 11. The proposed micromixer with baffles shows 1.5 times and 2.8 times the pressure drop in the micromixer with baffles at *Re* = 0.1 and 80, respectively. This indicates that the substantial pressure drop occurs at the baffle zones in the proposed design.

**Figure 12:**
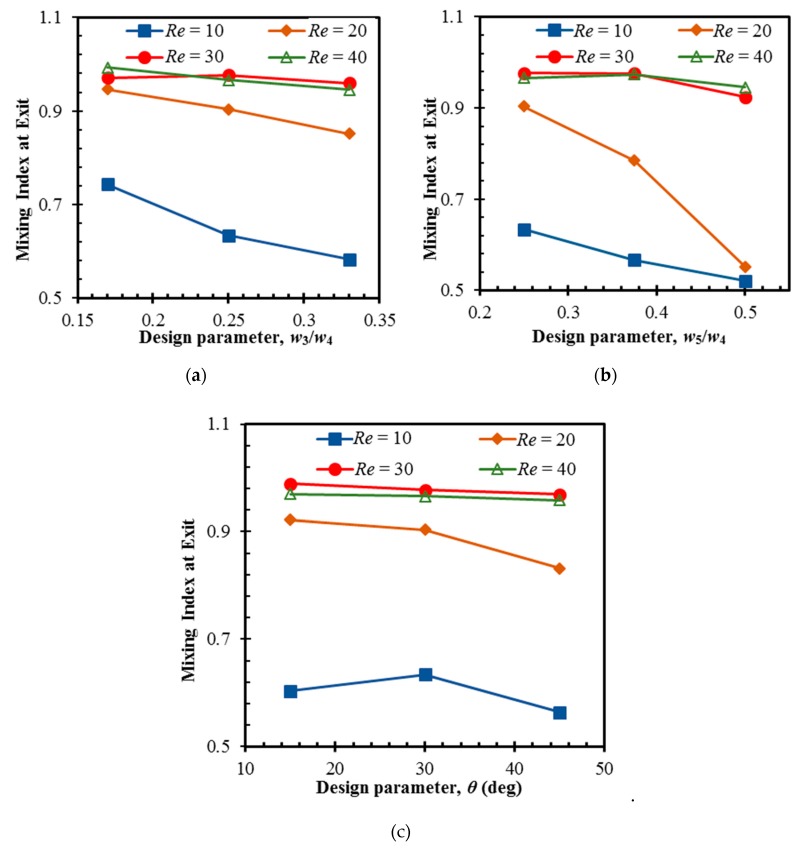
Effects of the design parameters on mixing: (**a**) *w*_3_*/w*_4_; (**b**) *w*_5_*/w*_4_; and (**c**) *θ*.

The response of the mixing index to the variation in the design parameters at different Reynolds numbers was explored through a parametric study, as presented in Figure 12. The ranges of the three dimensionless parameters, *w*_3_*/w*_4_, *w*_5_*/w*_4_, and *θ* (Figure 1), are listed in Table 3. The angle (*θ*) was varied with a fixed central baffle. Remarkable impacts of the design parameters on the mixing were not found at high Reynolds numbers of 30 and 40. The mixing index decreases rapidly with the change in parameters *w*_3_/*w*_4_ and *w*_5_/*w*_4_ at *Re* = 10 and 20. The change of *w*_3_/*w*_4_ causes the mixing index to vary by 27% and 11% within the tested range at *Re* = 10 and 20, respectively.

The largest change in flow area at the lowest ratio of *w*_3_/*w*_4_ = 0.17 causes strong separation vortices that enhance chaotic advection. Variations of 21% and 63% in the mixing index occur with the variation of w_5_/w_4_ at *Re* = 10 and 20, respectively. The narrow passage between the baffles and channel at the lowest ratio of *w*_5_/*w*_4_ causes local flow acceleration, which enhances the chaotic advection and mixing. The angle between the baffles has less impact on the mixing. Variations of 12% and 10% in the mixing index occur for the parameter *θ* at *Re* = 10 and 20, respectively.

## 5. Conclusions

A hybrid micromixer consisting of curved microchannels with radial obstructions and unbalanced SAR units was proposed to enhance the mixing in a wide range of Reynolds numbers. Numerical analyses of the mixing and flow were performed to evaluate the mixing indices at several Reynolds numbers of 0.1–80. The numerical results for the concentration distribution were validated by qualitatively comparing them with previous experimental results for a similar geometry.

In comparison with an earlier planar asymmetrical SAR micromixer, the present micromixer exhibited superior mixing performance over the whole Reynolds number range. For example, the mixing index was 221% higher at *Re* = 5. The proposed micromixer achieved mixing indices at the exit greater than 90% at *Re* ≥ 20 and a maximum mixing index of 99.8% at *Re* = 80. The advantage of introducing a mixing unit with radial baffles was confirmed through an increased rate of mixing development of the proposed micromixer in comparison with the P-ASAR mixer. At *Re* = 20, 63% of the reference microchannel length was sufficient to acquire a mixing index over 80%. The baffles enhanced the mixing index by 85%, 41%, and 23% at *Re* = 10, 20, and 40, respectively. The combined effects of the unbalanced collision, Dean vortices, and secondary flows because of the baffles contributed to the mixing in the proposed micromixer.

The parametric study indicated that the mixing index is sensitive to the parameters *w*_3_*/w*_4_ and *w*_5_*/w*_4_ at Reynolds numbers of 10 and 20, while the mixing is less sensitive to the angle *θ*. The results for *w*_3_*/w*_4_ and *w*_5_*/w*_4_ suggested that the maximum mixing index was obtained at the lower limit of each tested range (*w*_3_/*w*_4_ = 0.17 and *w*_5_/*w*_4_ = 0.25). The proposed micromixer shows high mixing performance in a wide Re range with 2D structure which has advantages in terms of fabrication and integration for microfluidic systems.

## Figures and Tables

**Table 1:** Parametric values of reference geometry.

Geometric Parameter	Value (in µm)
Width of major sub-channel, *w*_1_	200
Width of minor sub-channel, *w*_2_	100
Width of connecting-channel, *w*_3_	75
Width of main channel, *W*	300
Width of baffle channel, *w*_4_	300
Distance between the channel wall and baffle, *w*_5_	75
Radius of the curved channel, *R*	450
Angle between the baffles, *θ* (in degree)	30
Begining section length of channel, *L*_0_	500
Length of exit channel, *L*_e_	2950
Pitch length, *P*_i_	1200
Total length, *L*_t_	7950

**Table 2:** Calculation of uncertainty because of spatial discretization at *Re* = 40.

Parameter	Symbols	Values
Number of cells	*N*_1_/*N*_2_/*N*_3_	4.65 × 10^6^/3.15 × 10^6^/1.56 × 10^6^
Grid refinement factor	*r*	1.3
Computed mixing index (*N*_1_, *N*_2_, and *N*_3_)	*Φ*_1_ */Φ*_2_ */Φ*_3_	0.966/0.972/0.982
Apparent order	*p*	1.83
Extrapolated values	$\emptyset_{ext}^{21}$	0.957
Approximate relative error	$e_{a}^{21}$	0.62%
Extrapolated relative error	$e_{ext}^{21}$	0.94%
Grid convergence index	$GCI_{1,2}$	1.16%
	$GCI_{2,3}$	1.92%

**Table 3:** Design parameters and their ranges.

Parameter	Lower Bound	Upper Bound	Reference
*w*_3_ */w*_4_	0.17	0.33	0.25
*w*_5_ */w*_4_	0.25	0.50	0.25
*θ (degree)*	15	45	30

## Nomenclature

*c*	mass fraction
*D*	diffusion coefficient (m^2^/s)
*H*	total depth of main channel (µm)
*L_i_*	length of initial part of main channel (µm)
*L_e_*	exit channel length (µm)
*L_t_*	total length of the micromixer (µm)
*M*	mixing index
*M_0_*	mixing index at the exit
*N*	number of sampling points
*P*	pressure drop (Pa)
*Pi*	pitch length (µm)
*Re*	Reynolds number
*SAR*	split and recombine
*U*	average inlet velocity (m/s)
*W*	width of main channel (µm)
*w*_1_	width of major sub-channel (µm)
*w*_2_	width of minor sub-channel (µm)
*w*_3_	width of dislocation (µm)
*w*_4_	width of baffle channel (µm)
*w*_5_	gap between the channel wall and baffle (µm)
x, y, z	Cartesian coordinates
**Greek Symbols**	
*µ*	fluid dynamic viscosity (kg·m^−1^·s^−1^)
*ν*	fluid Kinematic viscosity (m^2^/s)
*ρ*	fluid density (kg/m^3^)
*σ*	standard deviation
**Subscripts**	
*i*	sampling point or fluid component
*m*	optimal mixing
max	maximum value
*x*	axial distance
